# Discovery of Protein–lncRNA Interactions by Integrating Large-Scale CLIP-Seq and RNA-Seq Datasets

**DOI:** 10.3389/fbioe.2014.00088

**Published:** 2015-01-14

**Authors:** Jun-Hao Li, Shun Liu, Ling-Ling Zheng, Jie Wu, Wen-Ju Sun, Ze-Lin Wang, Hui Zhou, Liang-Hu Qu, Jian-Hua Yang

**Affiliations:** ^1^RNA Information Center, Key Laboratory of Gene Engineering of the Ministry of Education, State Key Laboratory for Biocontrol, Sun Yat-sen University, Guangzhou, China

**Keywords:** long non-coding RNA, RNA-binding protein, GWAS, CLIP-Seq, RNA-Seq

## Abstract

Long non-coding RNAs (lncRNAs) are emerging as important regulatory molecules in developmental, physiological, and pathological processes. However, the precise mechanism and functions of most of lncRNAs remain largely unknown. Recent advances in high-throughput sequencing of immunoprecipitated RNAs after cross-linking (CLIP-Seq) provide powerful ways to identify biologically relevant protein–lncRNA interactions. In this study, by analyzing millions of RNA-binding protein (RBP) binding sites from 117 CLIP-Seq datasets generated by 50 independent studies, we identified 22,735 RBP–lncRNA regulatory relationships. We found that one single lncRNA will generally be bound and regulated by one or multiple RBPs, the combination of which may coordinately regulate gene expression. We also revealed the expression correlation of these interaction networks by mining expression profiles of over 6000 normal and tumor samples from 14 cancer types. Our combined analysis of CLIP-Seq data and genome-wide association studies data discovered hundreds of disease-related single nucleotide polymorphisms resided in the RBP binding sites of lncRNAs. Finally, we developed interactive web implementations to provide visualization, analysis, and downloading of the aforementioned large-scale datasets. Our study represented an important step in identification and analysis of RBP–lncRNA interactions and showed that these interactions may play crucial roles in cancer and genetic diseases.

## Introduction

Mammalian genomes encode thousands of long non-coding RNAs (lncRNAs) (Wang and Chang, 2011; Guttman and Rinn, 2012). lncRNAs play important roles in a variety of biological processes that have been implicated in regulating tumorigenesis through interaction with RNA-binding proteins (RBPs) (Konig et al., 2011; Wang and Chang, 2011; Guttman and Rinn, 2012; Ulitsky and Bartel, 2013). However, for the majority of lncRNAs, the mechanism underlying their interaction with RBPs remains unknown (Konig et al., 2011; Wang and Chang, 2011; Guttman and Rinn, 2012; Ulitsky and Bartel, 2013).

The control and function of lncRNA are governed by the specificity of RBPs (Wang and Chang, 2011; Guttman and Rinn, 2012). Increasing evidence suggests that many RBP–lncRNA interactions play important roles in correct transcriptional regulation (Konig et al., 2011; Wang and Chang, 2011; Guttman and Rinn, 2012; Ulitsky and Bartel, 2013). One emerging theme that many lncRNAs regulate gene expression by directing chromatin modificators to specific target regions (Ulitsky and Bartel, 2013). Significant fractions (20% in human) of lincRNAs are interacted with PRC2 and other chromatin-modifying complexes (Khalil et al., 2009; Guttman et al., 2011). The functional outcomes of some binding events have been revealed. For example, HOTAIR, which is transcribed from human HOX locus, guides repressor PRC2 to specific mammalian loci to silence gene expression and to promote cancer metastasis (Rinn et al., 2007; Wang et al., 2011). Besides, many lncRNAs have been shown to interact with other types of RBPs, including DNA methyltransferases (Schmitz et al., 2010; Di Ruscio et al., 2013), transcription factors (Wang et al., 2014), and splicing factors (Tripathi et al., 2010; Gong and Maquat, 2011; Yin et al., 2012). However, deciphering the interactions between hundreds of RBPs and thousands of lncRNAs remains a daunting challenge.

Genome-wide association studies (GWAS) have identified thousands of common genetic variants related to specific traits or disease phenotypes, and many of these variants (about 88%) lie in non-coding regions, which could potentially influence processing and expression of ncRNAs (Sethupathy and Collins, 2008; Hindorff et al., 2009; Ryan et al., 2010; Cabili et al., 2011; Kumar et al., 2013; Ning et al., 2014). For example, single nucleotide polymorphism (SNP) within miR-125a gene alters the processing of pri-miRNA by DGCR8 and causes recurrent pregnancy loss in a Han-Chinese population (Duan et al., 2007; Hu et al., 2011). Another study found that a papillary thyroid carcinoma-associated SNP, rs944289 affects the expression of lncRNA PTCSC3 by changing the binding activity of C/EBPα transcription factor (Cabili et al., 2011; Jendrzejewski et al., 2012). Although the genetic variants in interaction sites of RBP–lncRNA may interfere lncRNA functions and affected the susceptibility to human diseases, the relationships between genetic variants and interaction sites were yet unexplored.

Recent advances in high-throughput sequencing of RNA isolated by cross-linking immunoprecipitation (HITS-CLIP, CLIP-Seq, PAR-CLIP, CLASH, iCLIP) have provided powerful ways to identify RBP-associated RNAs and map such interactions in the genome (Chi et al., 2009; Hafner et al., 2010; Konig et al., 2011; Helwak et al., 2013; Fu, 2014; Fu and Ares, 2014). The application of CLIP-Seq methods has reliably identified Argonaute (Ago) binding sites and miRNA-target interactome (Chi et al., 2009; Hafner et al., 2010; Helwak et al., 2013). In fact, many more studies to date have been focused on understanding the function of RBPs in RNA metabolism (Konig et al., 2011; Fu, 2014), such as pre-mRNA splicing (Fu and Ares, 2014). While an increasing number of RBPs have been explored using CLIP technologies, binding peaks mapped to non-protein-coding genes have been routinely discarded and not further analyzed. However, this data will be a rich trove well worthy of mining RBP–lncRNA relationships.

In this study, we performed a large-scale integration of public RBP binding sites generated by high-throughput CLIP-Seq technology and identified thousands of RBP–lncRNA interactions. Furthermore, by combining GWAS and RNA-Seq data, we explored clinically relevant RPB–lncRNA interactions that may facilitate the translation of genetic studies of complex diseases into therapeutics.

## Materials and Methods

### Integration of RBP binding sites from published CLIP data

HITS-CLIP, PAR-CLIP, and iCLIP binding clusters/peaks data were retrieved from the gene expression omnibus and sequence read archive (SRA) (Barrett et al., 2013), the supplementary data of original references or directly from authors upon request. All binding sites coordinates were converted to hg19 and mm10 assemblies using the UCSC LiftOver Tool (Meyer et al., 2013).

### RBP target sites scanning in annotated lncRNA transcripts

Human gene annotations were acquired from GENCODE Version 17 (Harrow et al., 2012). Mouse gene annotations were extracted from Ensembl Gene Release 72 (Hubbard et al., 2009) and LiftOver to mm10 assembly. lncRNAs were further filtered to remove the transcripts overlapping with protein-coding genes. The aforementioned RBP CLIP clusters were used to intersect with the coordinates of all annotated transcripts to find their RBP binding sites, which were fed to Circos (Krzywinski et al., 2009) for visualization.

### TCGA tumor expression data and expression correlation of RBPs and lncRNAs

The Cancer Genome Atlas (TCGA) RNA-Seq expression datasets (level 3, IlluminaHiSeq_RNASeqV2) for 14 cancer types and gene annotation file (TCGA.hg19.June2011.gaf) were downloaded from TCGA Data Portal (Cancer Genome Atlas Research Network, 2008). Expression of 397 known lncRNAs can be measured in TCGA level 3 RNA-Seq data. Expression correlation (Pearson correlation coefficient) between lncRNAs and RBPs was estimated using co-expression program (the program is available from the authors upon request), which was written in C language and ALGLIB library, and *p*-value was adjusted with the false discovery rate (FDR) correction (Benjamini and Hochberg, 1995).

### Identification of disease-related SNPs in RBP binding sites associated with lncRNAs

Disease/phenotype associated SNPs were curated from published GWAS data provided by the NHGRI GWAS Catalog (Welter et al., 2014), Johnson and O’Donnell (2009), dbGAP (Mailman et al., 2007), and GAD (Becker et al., 2004). Additional SNPs in linkage disequilibrium (LD) with reported disease-related loci were selected with the criteria requiring an *r*^2^ value over 0.5 in at least one of the four populations (CEU, CHB, JPT, and YRI) genotype data of the HapMap project (release 28) (International HapMap 3 Consortium et al., 2010). For each SNP, rs ID were lifted to dbSNP build 141 based on the “RsMergeArch.bcp” and “SNPHistory.bcp” table from dbSNP, and genomic coordinates were lifted to the hg19 assembly using the UCSC LiftOver tool. All these disease-related SNPs or LD SNPs were mapped to exons and splicing sites (2 nt in the intron that is close to an exon) of the annotated lncRNA transcripts and further examined whether they were located in any RBP binding clusters.

### Data visualizations

RNA-binding protein–lncRNA interactions were deposited in our starBase V2.0 (Li et al., 2014) under the “Protein–RNA” section^1^. For each interaction, we provided links to our enhanced deepView genome browser^2^, which was written using a GD graphics library for PHP, to visualize RBP binding sites, lncRNAs, and other annotation tracks in an integrated display style similar to that of UCSC genome browser.

## Results

### The genome-wide binding map of RNA-binding proteins and the annotation of RBP–lncRNA interactions

**Figure 1:**
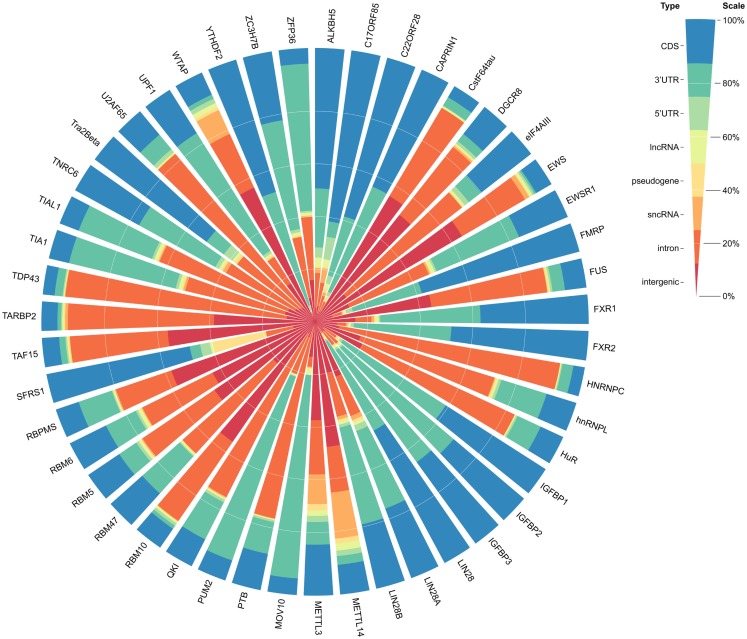
**The genomic context distributions of binding sites for 47 human RBPs**. Binding sites are mapped to genomic features in the following priority order: CDS, 3′UTR, 5′UTR, lncRNA, pseudogene, sncRNA, intron, intergenic.

We curated 117 published CLIP-Seq datasets to profile the genome-wide binding maps of 65 RBP. Unique binding sites of distinct RBPs varied from thousands to millions, and the genomic context distributions of binding sites for different RBPs distinguished from each other (Figure 1; Table S1 in Supplementary Material). For example, PUM2, a translational repressor during embryonic development and cell differentiation (Huang et al., 2011), predominately bound to 3′UTR regions of protein genes, while another translation inhibitor FMRP (Napoli et al., 2008) tended to interact with CDS. The discrepancy in binding context preferences for RBPs could root from different amounts of available datasets, usages of various variants of CLIP-Seq, varying sequencing depth, and/or genuine distinctions in the underlying recognition mechanism of RBPs.

**Figure 2:**
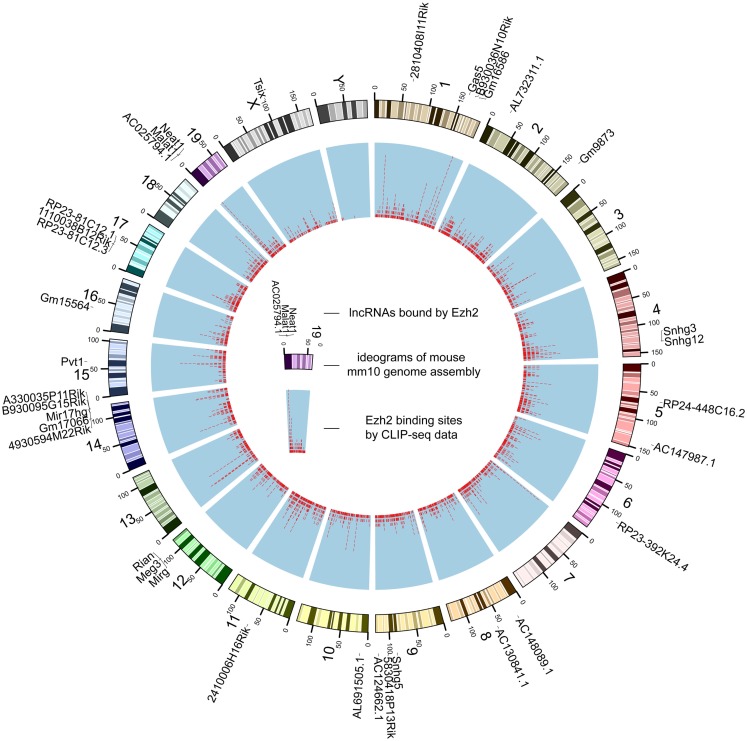
**The genome-wide binding map of Ezh2 in mouse**. The outer track is mouse chromosomes labeled with lncRNAs bound by Ezh2. The red tiles of the inner track represent the genomic coordinates of corresponding binding sites.

Despite that the majority of RBP binding sites were mapped to protein-coding genes, on average 1.1% of RBP binding sites lay within exons of human lncRNAs. In total, 21,073 and 1,662 RBP–lncRNA interactions were identified in human and mouse, respectively (Table 1). It is noteworthy that most well-studied lncRNAs interacted with chromatin modificators, acting as tethers or scaffolds (Khalil et al., 2009; Kung et al., 2013). Thus, we considered the binding features of Ezh2, a subunit of PRC2 complexes, by analyzing the CLIP-Seq data from mouse embryonic stem cells (Kaneko et al., 2013). Our results demonstrated that Ezh2 interacted with 35 lncRNAs including many imprinted RNAs, such as Tsix, Meg3, Rian, and Pvt1 (Figure 2), which was consistent with the epigenetic features of PRC2 (Zhao et al., 2010).

**Table 1:** **The summary of CLIP-Seq datasets used in this study and the resulting RBP–lncRNA interactions**.

Species	Experiments	RBPs	Cell lines/tissues	RBP binding sites mapped to lncRNAs	RBP–lncRNA interactions
Human	90	47	13	84,356	21,073
Mouse	27	18	20	5,330	1,662

### Exploring combinatorial effects among RBPs

**Figure 3:**
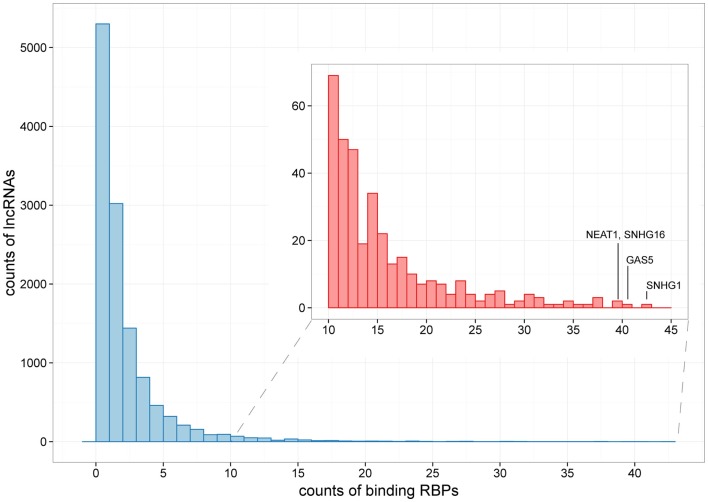
**The distribution of lncRNAs bound by different numbers of RBPs**. Histograms showing counts of lncRNAs bound by over 10 RBPs are zoomed in at the subpanel. SNHG1, GAS5, NEAT1, and SHNG16 are marked, which are bound by 42, 40, 39, and 39 RBPs, respectively.

**Figure 4:**
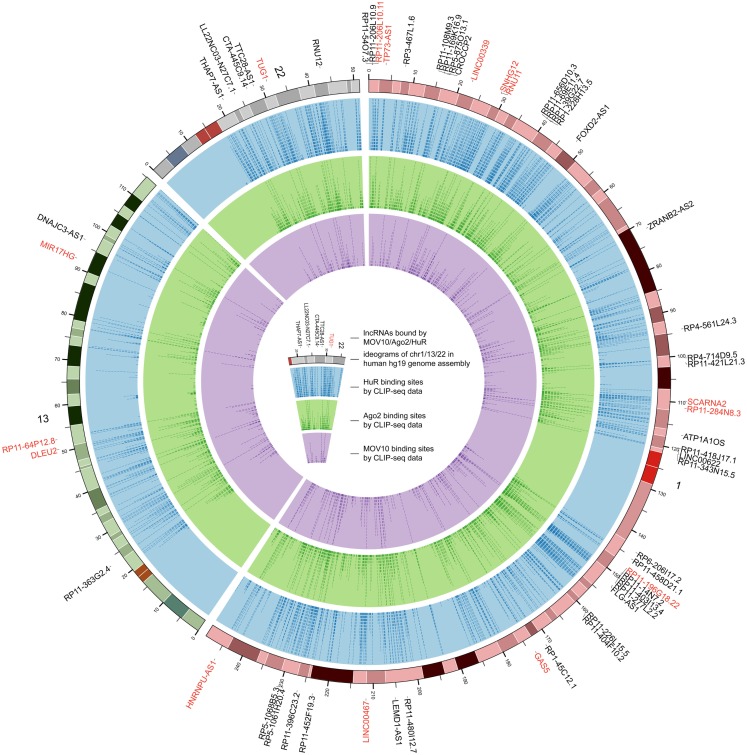
**The genome-wide binding map of HuR, Ago2, and MOV10 in human**. The outermost track represents ideograms of chr1, chr13, and chr22 in human genome. lncRNAs bound by these RBPs are labeled on the periphery, and those bound by at least two of the three RBPs at identical binding sites are colored red. The blue, green, and purple tracks indicate the binding positions of HuR, Ago2, and MOV10, respectively.

For the 12,255 human lncRNAs, 56.8% were found bound to at least 1 RBP. Surprisingly, 16 lncRNAs, including GAS5 and NEAT1, harbored binding sites of over 30 RBPs (Figure 3; Table S2 in Supplementary Material), indicating their diverse roles in biological processes when accompanied with different RBPs. Since one lncRNA could interact with multiple RBPs, it could be expected that some RBP binding sites were overlapped with each other. Therefore, we explored combinatorial effects among RBPs by employing integrated CLIP-Seq datasets. For example, we utilized PAR-CLIP data generated in HEK293 and intersect binding sites of three RNA destabilizer HuR, Ago2, and MOV10. The results showed that tens of lncRNAs, including cancer-related lncRNAs TUG1, DLEU2, and GAS5, were bound by at least two of the three RBPs at identical binding sites (Figure 4). This phenomenon suggested that the stabilities of these lncRNAs were likely under joint control of these three RBPs, which could be explained by their confirmed interplays in HEK293 (Chendrimada et al., 2007) and Hela cells (Kim et al., 2009).

### Expression association of RBP–lncRNA interactions

**Figure 5:**
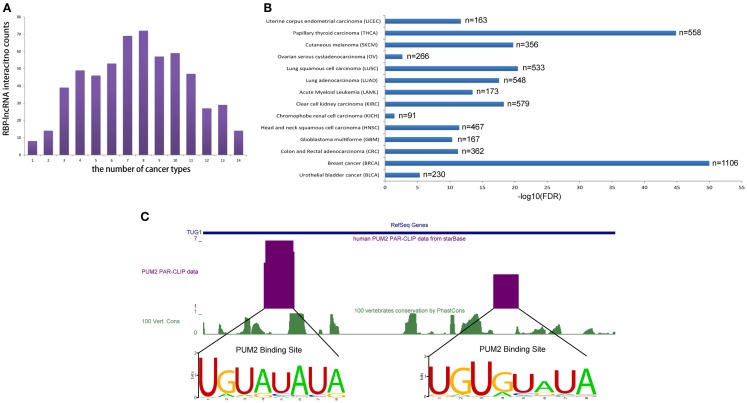
**RBP–lncRNA interactions are supported by co-expression analysis in 14 types of cancers**. **(A)** Histograms show RBP–lncRNA interactions with expression association (Pearson correlation, *p* < 0.05) in at least one cancer type. **(B)** The expression levels of PUM2 and TUG1 are positively correlated (*p* < 0.05) in all 14 cancers. **(C)** The PUM2 binding sites on TUG1 are inferred from PAR-CLIP data, and the consensus recognition motif UGURUAUA are conserved in mammals.

To realize the roles of RBP–lncRNA interactions in cancer, we preformed co-expression analysis between RBPs and lncRNAs by virtue of 90 human CLIP-Seq datasets and expression data from more than 6,000 tumor samples in 14 types of cancer. Up to 583 pairs concerning 47 RBPs and 49 lncRNAs showed strong correlation at expression levels in at least 1 cancer type (Figure 5A). Marvelously, PUM2 and TUG1 involved with cell cycle regulation (Khalil et al., 2009; Huang et al., 2011) showed significant positive expression correlation (*p* < 0.05) in all 14 cancer types (Figure 5B). Two potential PUM2 binding sites on TUG1 have the consensus recognition motif UGURUAUA, which was highly conserved in mammals (Figure 5C).

### Predicting GWAS-associated RBP binding sites in lncRNAs

Although GWAS over the years have revealed a significant number of genetic variants related to diseases or phenotypes, a considerable portion of these identified loci are not within protein-coding genes and therefore not functionally explained to date (Hindorff et al., 2009). Here, we tried to fill this gap by connecting RBP binding sites in lncRNAs and potential disease-related SNPs.

Altogether, 87,677 unique disease-related SNPs were collected from four public GWAS data source (Table S3 in Supplementary Material, detailed in Section “Materials and Methods”). Considering that additional SNPs in LD with reported disease-related loci may also map to RBP binding sites in lncRNAs, we perform LD analysis to extracted SNPs that had high LD relationship with disease-related SNPs using a threshold of *r*^2^ > 0.5 in at least one population from the HapMap CEU, CHB, JPT, and YRI genotype data, which yielded a total of 895,968 disease-related or LD SNPs.

**Figure 6:**
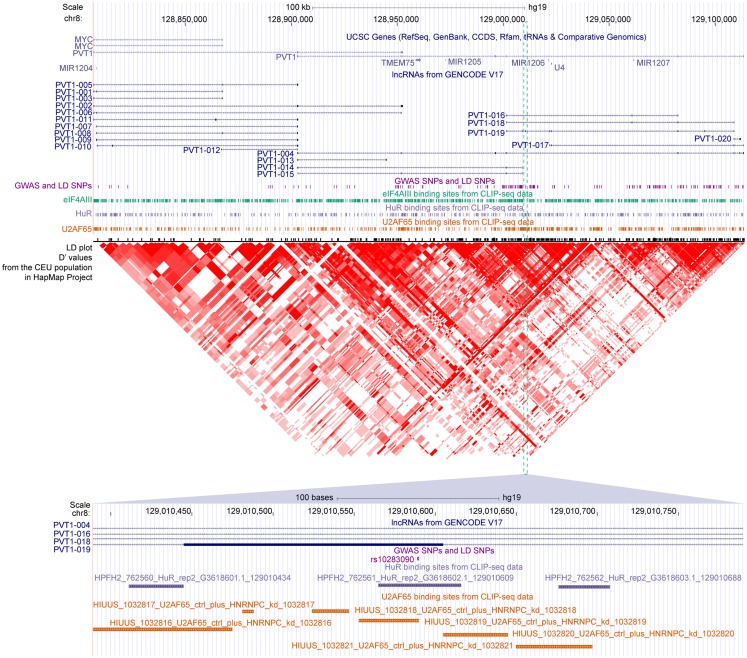
**The GWAS-associated SNPs and binding sites of three RBPs in the locus of PVT1**. Gene annotations from UCSC, lncRNAs from GENCODE, GWAS, and LD SNPs, binding sites of eIF4AIII/HuR/U2AF65 and LD plot from HapMap are shown accordingly. The SNP rs10283090 overlapped with binding sites of HuR and U2AF65 are zoomed in at the bottom panel.

We found that 2431 of these SNPs were mapped to the exons of 2089 transcripts of 1489 lncRNA genes, among which 162 SNPs were also located in at least 1 binding sites of 29 RBPs (Table S4 in Supplementary Material). For example, three disease-related SNPs, namely, rs16902485, rs10283090, and rs2720659, resided in the exons of lncRNA PVT1. According to the GWAS annotations of Johnson and O’Donnell (2009), the latter two of the three SNPs were associated with “type II diabetes mellitus,” which was in good accordance with the recent reports showing that PVT1 may contribute to diabetic nephropathy (Hanson et al., 2007; Alvarez and DiStefano, 2011; Alwohhaib et al., 2014). These SNPs were also overlapped with binding sites of U2AF65, HuR, and eIF4AIII, respectively (Figure 6), suggesting variants in these sites might result in impaired binding of these RBPs to PVT1, which thereby might lead to the development of corresponding diseases.

Next, we checked whether disease-related SNPs might be located in the splicing sites of lncRNAs and affect the alternative splicing of lncRNAs. We defined a splicing site as the 2 nt within an intron close to the exon–intron junction. As a result, we found that only 24 SNPs lay within lncRNA splicing sites (Table S4 in Supplementary Material), among which only 1 SNP, rs17207481, was overlapped with binding sites of FUS and HuR. These results suggested that SNPs exerted limited effects on disease occurrence through the mechanism of disturbing alternative splicing of lncRNAs.

### Comparative analysis of RBP targets using the deepView genome browser

**Figure 7:**
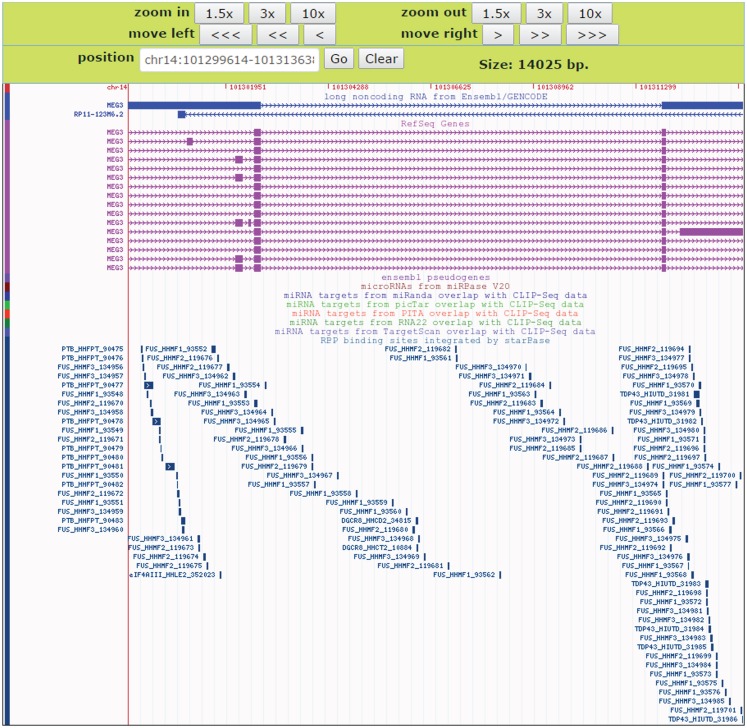
**An instance for displaying RBPs target sites in the deepView Browser of starBase V2.0**. The predictive FUS binding sites on MEG3 are visible in the RBP binding sites track. In this track, the binding sites of other RBPs such as TDP-43 and PTB on MEG3 are also showed, which facilitates comparative analysis of binding events of multiple RBPs.

To facilitate comparative analysis of the CLIP-Seq datasets and exploration of RBP–lncRNA interactions, we developed the improved deepView Genome Browser^2^ in starBase V2.0 (Li et al., 2014). In the query page of the browser, users can input one interested genomic region in the “search term” and select corresponding genome assembly to gain an integrated view of various genomic features. Information on binding sites of RBPs, predicted miRNA-target sites overlapped with CLIP-Seq data, as well as gene annotations from RefSeq and Ensembl were provided in toggleable tracks. The image of the browser will be updated immediately by clicking the “refresh tracks” button when users change track options. Figure 7 illustrated the visualization of FUS–MEG3 interactions with deepView. Users can click the “zoom in” or “zoom out” button at the top to shrink or extend on the center of the annotation tracks window by 1.5-, 3-, or 10-folds. Clicking the “View region at UCSC” button will redirect users to the UCSC page and exhibit the current region on the UCSC genome browser. To explore RBP binding sites on a particular gene, users can type its gene symbol in the position textbox and then click the “GO” button to update the display image to determine, which RBPs might participate in regulating the gene. Our visualization method allows a direct comparison of binding patterns of different RBPs, binding preferences of one particular RBP in different cell lines and tissues, and genomic contexts of RBP binding sites.

## Discussion

Although a few dozen lncRNAs have been characterized to some extent and reported to function in important cellular processes, the functions of most annotated lncRNAs are unknown (Guttman and Rinn, 2012; Ulitsky and Bartel, 2013). Several bioinformatics resources and tools have made efforts to functionally annotate lncRNAs (Da Sacco et al., 2012), such as fRNAdb (Kin et al., 2007) and ncFANs (Liao et al., 2011). These tools mainly inferred lncRNA function by their differential expression in distinct biological states or their co-expression patterns with protein-coding genes, but little attention was paid to the relationship of lncRNAs and their bounded proteins. In this study, by analyzing a large set of RBP binding sites derived from all available CLIP-Seq experimental techniques (PAR-CLIP, HITS-CLIP, iCLIP, CLASH), we have shown extensive and complex RBP–lncRNA interaction networks (Figure 1).

Recent studies have revealed that many lncRNAs function through specific interactions with RBPs, but whether these interactions are direct and specific remains controversial. RBP–lncRNA interactions identified by low stringent immunoprecipitation of non-cross-linked RNA–protein complexes, such as RIP-Chip and RIP-Seq, may contain indirect binding relationships (Konig et al., 2011). In comparison to previously reported significant fractions (10% in mouse) of PRC2-associated lncRNAs (Zhao et al., 2008), we found that a relatively small fraction (~1%) of lncRNAs were bound by Ezh2 in mouse (Figure 2). Therefore, we provide enhanced resolution to determine lncRNA functional networks based on RBP–lncRNA interactions supported by high-throughput CLIP-Seq data. More than 80,000 binding clusters identified from 65 different RBPs represent a valuable resource for resolving some obstacles that have arisen in efforts to understand lncRNA action. Nevertheless, although CLIP-Seq is designed to detect direct binding events of proteins and RNAs, the resulting data might still contain false positives and false negatives, which may root from every cumbersome step of this technique. To minimize the impact of such false discoveries, we filtered the origin results by the reported FDR and provided evidences such as number of CLIP reads and number of supporting experiments, which may help users to gain RBP–lncRNA interactions of high-confidence.

By cross analysis of binding maps for multiple RBPs, this study offers a new resource to understanding joint control of target lncRNA expression. While only 65 RBPs were analyzed, we found that many of the RBPs bound to the same lncRNA (Figure 3). This is consistent with the compelling idea that lncRNAs can serve as scaffolds that assemble many relevant RBPs to regulate gene expression (Wang and Chang, 2011; Ulitsky and Bartel, 2013). At the same time, we also identified hundreds of identical binding sites that bound by multiple different RBPs in lncRNAs (Figure 4), probably reflecting competition among RBPs that binding on a given lncRNA.

Our combined analysis of CLIP-Seq data and GWAS data revealed hundreds of disease-related SNPs resided in the RBP binding sites of lncRNAs (Table S4 in Supplementary Material). Unlike the sporadic attempts on simply finding genetic variants associated with disease susceptibility within lncRNA genes (Bochenek et al., 2013; Mirza et al., 2014), our approaches focused on SNPs that might impact on the binding events between RBPs and lncRNAs. Since most lncRNAs fulfill their roles through by forming complex with their protein partners, our results provide insights on the functions of lncRNAs from the perspective of RBP binding malfunction in diseases, which in turn may contribute to disease etiology.

Overall, our studies and the accompanying datasets demonstrated that one single lncRNA will generally be bound and regulated by one or multiple RBPs, the combination of which may coordinately determine the final regulatory outcome. We have also shown that an exhaustive and high-resolution RBP–lncRNA interaction map will help to discover genetic variations that contribute to complex genetic diseases by affecting post-transcriptional gene regulation.

## Author Contributions

Jian-Hua Yang, Liang-Hu Qu, and Jun-Hao Li conceived the project. Jun-Hao Li, Shun Liu, Ling-Ling Zheng, and Jian-Hua Yang performed the computational and statistical analysis. Jun-Hao Li, Shun Liu, Ling-Ling Zheng, Jian-Hua Yang, Liang-Hu Qu, Jie Wu, Wen-Ju Sun, Ze-Lin Wang, and Hui Zhou wrote the manuscript. Liang-Hu Qu and Jian-Hua Yang supervised the project. All authors read and approved the final manuscript.

## Conflict of Interest Statement

The authors declare that the research was conducted in the absence of any commercial or financial relationships that could be construed as a potential conflict of interest.

## Supplementary Material

The Supplementary Material for this article can be found online at http://www.frontiersin.org/Journal/10.3389/fbioe.2014.00088/abstract

Table S1 **The summary of binding sites distribution across genomic features for 47 human RBPs**.

Table S2 **Counts of binding RBPs in the 12,255 human lncRNAs**.

Table S3 **Disease/trait related SNPs collected from four public GWAS databases**.

Table S4 **Disease/trait related SNPs overlapped with RBP binding sites in lncRNAs**.

## Footnotes

^1^http://starbase.sysu.edu.cn/rbpLncRNA.php

^2^http://starbase.sysu.edu.cn/browser.php
